# Supplementary material for: Prior information differentially affects discrimination decisions and subjective confidence reports
Source: Nat Commun. 2023 Sep 6;14:5473. doi: 10.1038/s41467-023-41112-0 (PMC10482953; doi:10.1038/s41467-023-41112-0)
Supplement: Supplementary file 1 — Supplementary Information [file 41467_2023_41112_MOESM1_ESM.pdf]

Supplementary Information

for "*Prior information differentially affects discrimination decisions and subjective confidence reports*"

Authors: Marika Constant[1,2,3], Michael Pereira[4], Nathan Faivre[4], Elisa Filevich[1,2,3,5]

[1] Humboldt-Universität zu Berlin, Faculty of Life Sciences, Department of Psychology, Unter den Linden 6, 10099 Berlin, Germany

[2] Bernstein Center for Computational Neuroscience Berlin, Philippstraße 13 Haus 6, 10115 Berlin, Germany

[3] Berlin School of Mind and Brain, Humboldt-Universität zu Berlin, Luisenstraße 56, 10115 Berlin, Germany

[4] Université Grenoble Alpes, Université Savoie Mont Blanc, CNRS, LPNC, 38000 Grenoble, France

[5] Hector Institute for Education Sciences & Psychology, University of Tübingen, Europastraße 6, 72072, Tübingen, Germany

Corresponding author: Marika Constant, marika.constant@gmail.com

Contents

Experiment 2 Behavioural Results **2**

Supplementary Methods **2**

Flexible Model Definition *2*

Model Fitting *3*

Model Simplification *5*

Model Recovery *7*

Flexible Model - Experiment 3 .... *9*

Model Adaptations 9

Fitting Metacognitive Noise 10

Supplementary Discussion **11**

Alternative Explanations *11*

Metacognitive Noise 11

Lognormal Metacognitive Noise 13

Translation Noise 14

Confirmatory Evidence Accumulation 14

Motion Aftereffects 15

Confidence Leak 16

Prior Incongruence Analysis 18

Supplementary References **21**

Experiment 2 Behavioural Results

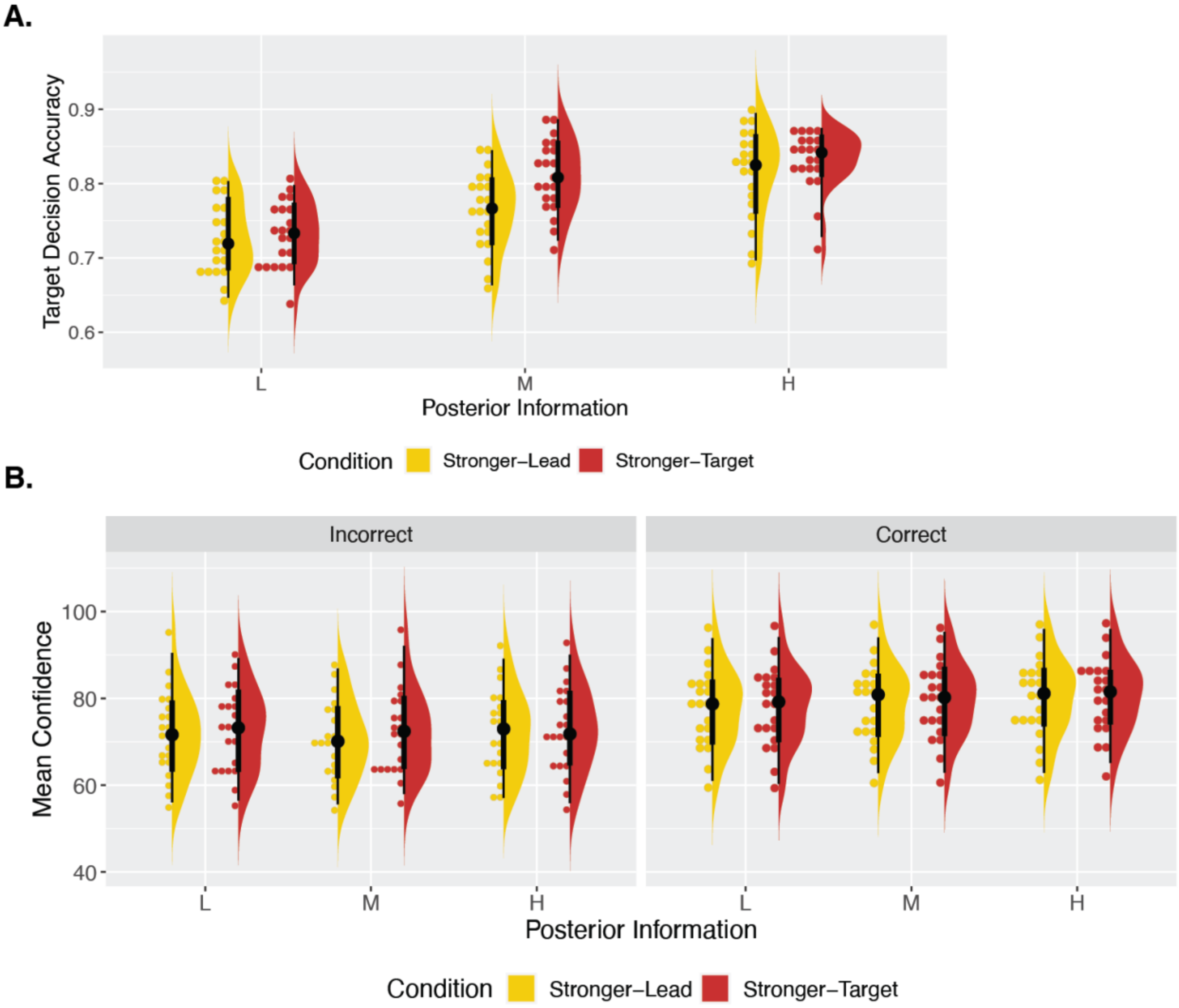

Figure S1. Experiment 2 Behavioural Results. A. Effect of condition on accuracy. We found a significant interaction between the effects of condition (which stimulus was stronger) and posterior level on target decision accuracy. Decision accuracy was higher in the Stronger-Target condition and this effect was the strongest at the medium posterior level, suggesting participants to underweight prior information in their decisions, as in Experiment 1. In the raincloud plots, the right-half, split violin plots show the probability density, and vertical black lines show the median, IQR, hinges showing the first and third quartiles, and vertical whiskers showing +/- 1.5IQR. The binned dotplots on the left half show each individual subject (N=25) as a point. **B. Effect of condition on confidence.** We found a significant effect of condition on confidence, with higher confidence in the Stronger-Target condition (N=25 participants). Source data are provided as a Source Data file.

Supplementary Methods

Flexible Model Definition

In the model, the lead stimulus, s_{lead}, generated an internal signal, r_{lead}, by adding normally distributed internal noise, $\mathcal{N}(0, \sigma_{\text{prior}})$. The lead decision was made by comparing this

internal signal to the decision criterion – signals to the right of the criterion led to a rightward decision, and signals to the left led to a leftward decision. This lead decision then also led to an internal confidence value according to:

$$conf_{lead} \;=\; \Phi(\frac{|r_{lead}|}{b * \sigma_{prior}})$$

where b is the confidence bias, captured by a general over- or underestimation of the signal variance. The strength of the rightward prior in the target decision was then equal to this $conf_{lead}$, weighted by the weighting parameter at the decision level, w_{choice}:

$$p(R)_{choice} \;=\; \Phi(\frac{|r_{lead}|}{b\ w_{choice}\ \sigma_{prior}})$$

The target stimulus, which was rightward if the lead decision was correct and leftward if not, then generated an internal signal, r_{target}, by adding normally distributed internal noise, $\mathcal{N}(0, \sigma_{likelihood})$. The target decision was based on the posterior probability of a rightward target stimulus, which was determined by the integration of the likelihood and the prior. As shown previously by Lisi et al.[1], this was computationally equivalent to shifting the target decision criterion and comparing r_{target} to that shifted decision criterion:

$$\theta \;=\; -\frac{\sigma_{likelihood}}{w_{choice}\sigma_{prior}}|r_{lead}|$$

Then, explicit confidence in the target decision was computed as the perceived posterior probability correct, given the evidence and decision. The posterior was again the product of the likelihood and weighted prior, but now the prior was weighted according to the weighting parameter at the confidence level, w_{conf}:

$$p(R)_{conf} \;=\; \Phi(\frac{|r_{lead}|}{b\ w_{conf}\ \sigma_{prior}})$$

to give:

$$conf_{right} \;=\; \frac{p(R)_{conf}\ p(r_{target}|R)}{(1-p(R)_{conf})(1-\ p(r_{target}|R)) + p(R)_{conf}\ p(r_{target}|R)}$$

$$= \frac{p(R)_{conf}\ (1 + erf\left[\frac{r_{target}}{b\ \sigma_{likelihood}\sqrt{2}}\right])}{1 + \left(2p(R)_{conf} - 1\right)erf\left[\frac{r_{target}}{b\ \sigma_{likelihood}\sqrt{2}}\right]}$$

This was the predicted confidence (model_conf) following rightward decisions and $conf_{left}$ = (1-$conf_{right}$) was model_conf following leftward decisions.

Model Fitting

We fit each model using 6000 effective samples across 3 chains, as well as 2000 burn-in samples per chain. All initial parameter values were sampled from a uniform distribution between 0.5 and 1.5. We ensured that all R-hat values were lower than 1.1, indicating good

convergence. Lognormal priors were set on the group mean parameters - $w_{choice\_}mu$, $w_{conf\_}mu$, and b_mu, and subject-wise parameters w_{choice}, w_{conf} and b were modelled as being normally distributed around group mean parameters. Target decisions were modelled as being Bernoulli distributed. Following the procedure used in previous work with a similar modelling approach[2], we allowed for a small degree of noise (σ=0.0125) between the predicted confidence outlined above (model_conf) and the observed confidence ratings, accounting for some imprecision of participants' confidence ratings. Posterior predictive checks revealed the model to well capture choice probabilities, as well as differences between conditions in confidence (Figure S2), although confidence was predictably underestimated due to the simplification, explained below.

Priors:

$w_{choice\_}mu \sim \text{Lognormal}(0,0.5)$

$w_{conf\_}mu \sim \text{Lognormal}(0,0.5)$

$b\_mu \sim \text{Lognormal}(0,0.5)$

$w_{choice\_}sd \sim \text{Lognormal}(0,0.5)$

$w_{conf\_}sd \sim \text{Lognormal}(0,0.5)$

$b\_sd \sim \text{Lognormal}(0,0.5)$

Model:

$w_{choice} \sim \mathcal{N}(w_{choice\_}mu\ ,\ w_{choice\_}sd)$

$w_{conf} \sim \mathcal{N}(w_{conf\_}mu\ ,\ w_{conf\_}sd)$

$b \sim \mathcal{N}(b\_mu\ ,\ b\_sd)$

$\text{choice} \sim \text{bernoulli}(\Phi_{\text{right}})$

$\text{conf} \sim \mathcal{N}(\text{model\_conf},\ 0.0125)$

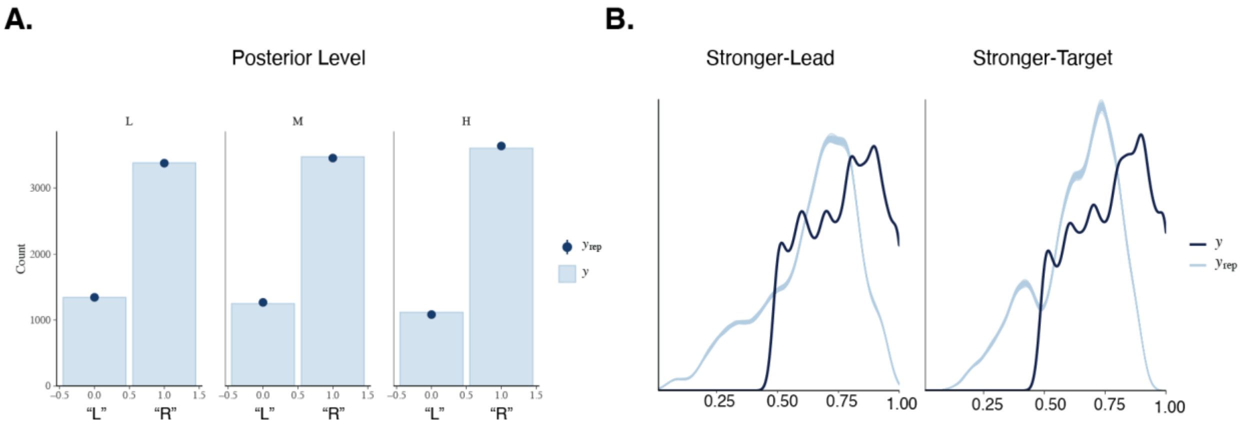

Figure S2. Posterior Predictive Checks. A. Modelling discrimination decisions. The light blue bars (y) show the true choice probabilities per posterior level in the data, with rightward target decisions shown at 1, and leftward target decisions shown at 0. The dark blue points (y_{rep}) reflect the predicted choice probabilities from the model, generated by sampling from the posterior distribution and simulating target decisions from those samples. **B. Modelling confidence.** In dark blue are the kernel density distributions of confidence in the data per condition (y). In light blue are the predicted kernel density

distributions for confidence per condition from the model (y_{rep}), generated by sampling from the posterior distribution and simulating target decisions and then confidence from those samples. These are split by the condition to demonstrate that, although the model predictably underestimates confidence, y_{rep} is shifted relative to y similarly in both conditions, therefore allowing us to capture differences in conditions, and this is our primary interest. Source data are provided as a Source Data file.

Model Simplification

The Φ_{right} and model_conf values were computed as outlined in the main text, however, instead of fitting internal signal values r_{lead} and r_{target} on every trial, which would lead to an overparameterized model and complexity issues in the sampling procedure, we simplified the model by fitting confidence based on the external stimulus values. The external stimulus values reflect the mean of the internal signal distribution. We still used the internal noise in the model as otherwise specified. The choice probability was computed by taking the integral across all possible r_{lead} values, weighted by their likelihood, and hence was not impacted by this simplification. For confidence however, fixing the internal signals at the means meant that the model underestimated confidence in expected ways, particularly following incorrect choices. In this model, stimuli would always lead to internal samples on the correct side of the decision criterion. The model would then assign a confidence value *c* above 50% to these correct choices, and consequently *1-c* to the alternative, incorrect choice. On incorrect trials, this is then incompatible with participants' ratings, which were bounded at 50% due to the scale, so the model must then adjust the confidence bias parameter to increase confidence predictions on incorrect trials. This, in turn, decreases confidence predictions for correct trials (as confidences in both choice alternatives must sum to 1), causing a trade-off between the model's ability to account for confidence on incorrect trials and on correct trials. This forces an underestimation of confidence overall. The simulated results from this fit simplified hierarchical model are shown in Figure S3, with the underestimated confidence apparent in Figure S3B, despite the differences between conditions still being well captured.

This primarily impacted the confidence bias parameter *b*, and because this was particularly strong following incorrect trials, *b* was skewed to be inflated in order to increase the value of $conf_{Incorrect}$, and in turn decrease the value of confidence in the correct option ($conf_{Correct}$) which is equal to 1-$conf_{Incorrect}$. Admittedly, this simplification affected not only the bias parameter *b* but also slightly impacted w_{conf}. However we note that it had an effect in a direction directly against our results, as it increased w_{conf}.. We confirmed this with a parameter recovery analysis in which we simulated data with free internal signals but fit the simplified model, which led to slightly inflated recovery of w_{conf} (Figure S4). Hence, if we assume participants to also have internal signals, we can expect their true w_{conf} values to be slightly lower than the values we fit, which is even further in line with our finding that w_{conf} was lower than w_{choice}.

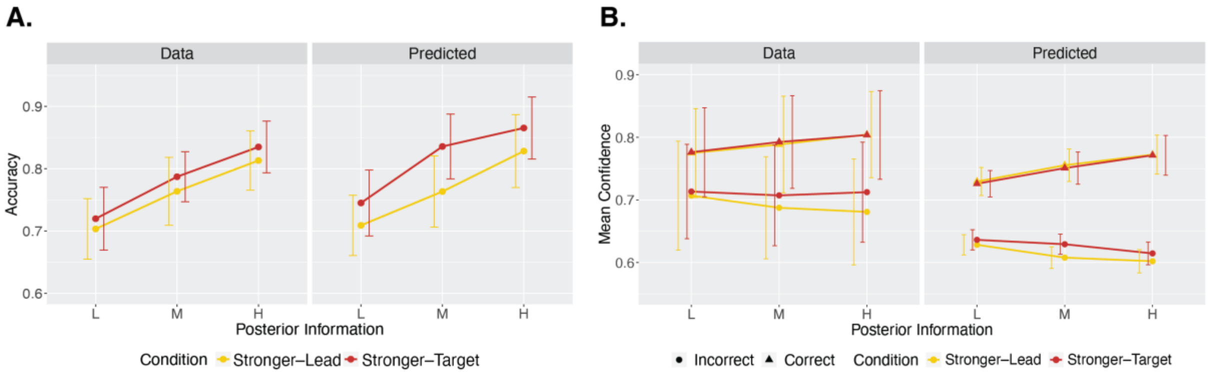

Figure S3. Simplified Model Results Against Data. A. Observed vs predicted response accuracy. The left panel shows the mean observed accuracies per posterior level and condition (N=20 participants). The right panel shows the predicted accuracies generated from sampling the fit posterior group mean parameter distributions 1000 times, and simulating 720 trials per participant for each of those sampled parameters. Note that we use the sample group mean parameter for simulating trials, but still used each participant's staircased coherences, internal noise and decision bias. Error bars capture standard deviation (SD) of accuracies across participants (N=20). **B. Observed vs predicted confidence ratings.** On the left are the data, showing mean confidence following correct and incorrect decisions per posterior level and condition (N=20 participants). On the right is mean confidence generated from sampling the fit posterior parameter distributions 1000 times and simulating 720 complete trials per participant based on those parameters. Note that we use the sample group mean parameters for simulating trials, but still used each participant's staircased coherences, internal noise and decision bias. The error bars capture standard deviation (SD) of the mean confidence across participants. Source data are provided as a Source Data file.

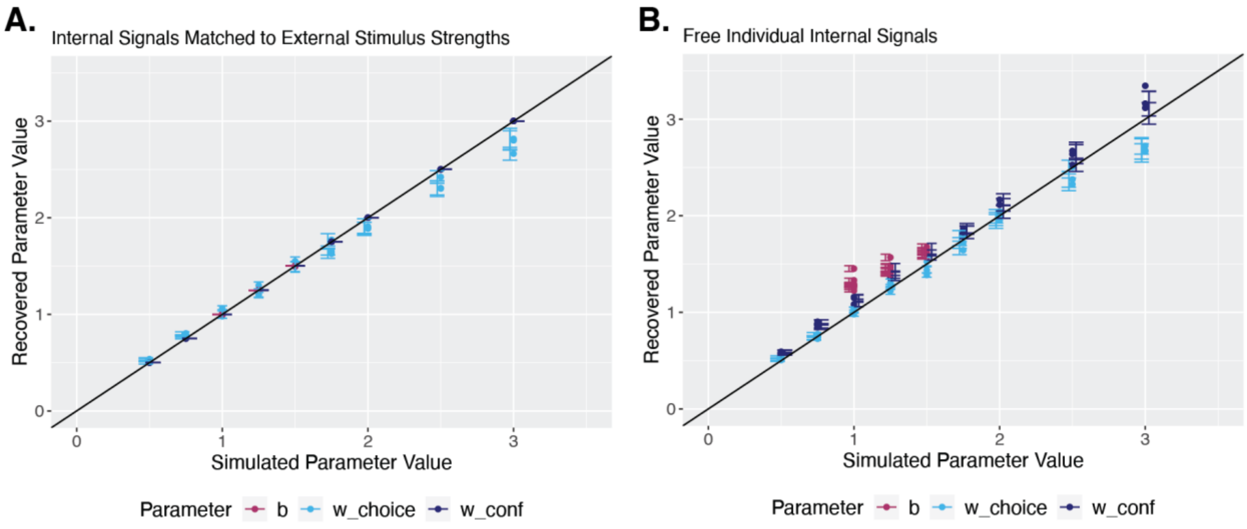

Figure S4. Parameter Recovery Analyses. A. Fixed internal signals (simplified model). Parameter recovery after simulating from the simplified model. The black line indicates perfect parameter recovery. This reveals good parameter recovery of the model when data is simulated from it. Points reflect the mean recovered parameter values across 20 repetitions of simulating data and fitting the model, and error bars reflect SEM across these repetitions. **B. Free internal signals.** Parameter recovery after simulating internal signals but then fitting with the simplified model. This reveals parameters to be reasonably well recovered, despite the simplification. The largest impact is on recovery of *b*, which is inflated relative to the simulated confidence bias. The simplification also leads to a slightly inflated recovery of w_{conf}, but this works directly against our conclusion that w_{conf} is smaller than w_{choice}, and therefore that the prior is weighted more strongly in confidence than in the decision. Points reflect the

mean recovered parameter values across 20 repetitions of simulating data and fitting the model, and error bars reflect SEM across these repetitions. Source data are provided as a Source Data file.

We also include a model in which we forced any confidence below 0.5 to be set to 0.5, the minimum of the scale, to prevent the skewing of parameters, and that model confirms these results (Figure S5), although there were some convergence challenges when introducing the lower confidence bound.

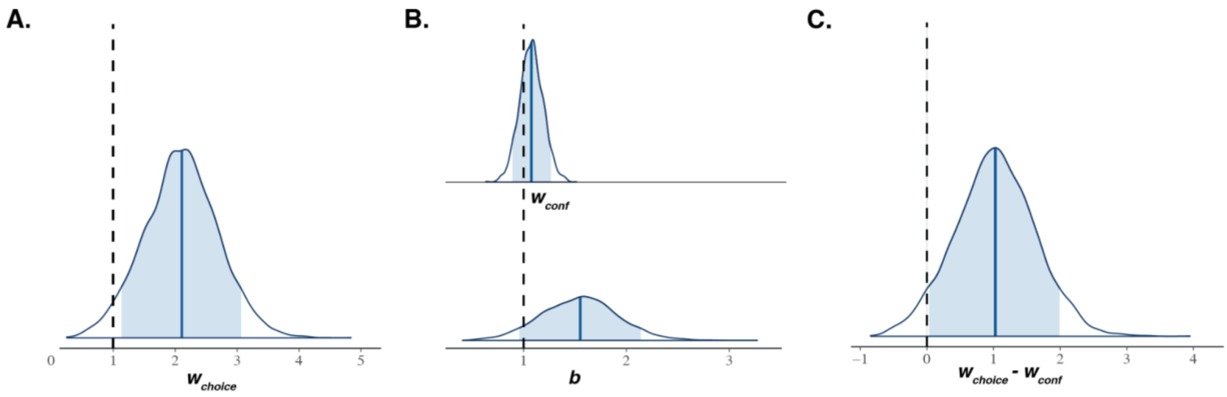

Figure S5. Bounded Confidence Model Results. In the bounded confidence model, everything was the same as in the Flexible Model except that any predicted confidence below 0.5 was set to 0.5, which was the minimum of the scale provided to participants. This prevented skewing of parameters due to the model simplification, described above. However, there were some convergence issues due to the discontinuity this introduced. All group parameters converged (R-hat < 1.1) but four individuals within the model had parameters that did not converge. We were, however, able to fit the model individually, and then it converged for all except one participant, and led to similar estimates as in the hierarchical fit. **A. Posterior distribution for w_{choice}.** The posterior distribution for the group mean parameter of the weighting of prior information in the decision, w_{choice}. The blue shaded region shows the 89% credible interval and the vertical black dashed line reflects optimal weighting of the prior in the decision (w_{choice} = 1). **B. Posterior distribution for w_{conf} and *b*.** The top posterior distribution is for the group mean parameter of w_{conf}. The lower posterior distribution is for the group mean parameter of *b*. The blue shaded regions show the 89% credible intervals and the vertical black dashed line corresponds to the parameter values of an optimal observer. Note that the estimates for both of these parameters are lower here in comparison to the unbounded version of the model, as this helps reduce the skew due to the model simplification. **C. Posterior Group Difference Distribution of w_{choice} - w_{conf}.** The posterior distribution for the difference in the group mean parameters w_{choice} and w_{conf}. The blue shaded region shows the 89% credible interval and the vertical black dashed line reflects no difference in the two parameters (w_{choice} - w_{conf} = 0). 0 is excluded from the 89% credible interval, suggesting w_{choice} and w_{conf} to be credibly different from one another. Source data are provided as a Source Data file.

Model Recovery

In order to ensure that our models were adequately distinguishable from one another, we performed a model recovery analysis in which we simulated data from parameters that were associated with the different models, and checked whether the correct models were recovered. We simulated data from each of the simpler models, as well as two parameter combinations of the Flexible Model, one case with w_{choice} greater than w_{conf}, and one with that

reversed, and then compared the model fits in each case using the PSIS-LOO CV approach[3]. This was repeated 10 times for every model/parameter combination. We set the criterion for the winning model to be that with the highest ELPD, and we considered this to be conclusive if the ELPD was higher by at least 4, and with a magnitude of difference that was at least 2 times the standard error of the difference. Results of this analysis are shown in the confusion matrix below (Figure S6A), which shows for each true generative model, the proportion of the 10 repetitions of this recovery analysis in which each competing model was the winning one. This reveals a good model recovery accuracy of 95%. However, not all of those results are conclusive according to the above criteria (Figure S6B). This was not surprising, given our models. We expected that, when fitting data simulated from the Equal Model, both the Flexible and Equal Model would be able to predict the data, since the Flexible Model is able to give equal w_{choice} and w_{conf} parameters as well. When data were simulated from the Optimal Model, we expected the Flexible, Equal, and Optimal Models to all predict the data well, since all three models can account for w_{choice} and w_{conf} values of 1. So, while we still show that the winning model is almost always correct, we did not expect this to be conclusive for the Equal or Optimal models. The most critical test for us was that the Flexible Model was only the conclusively winning model when the data came from that model. This indicates that it is distinguishable from the simpler models, in that it would only win the comparison when data was truly simulated from that model, and hence w_{choice} and w_{conf} were different from optimal, different from each other, and different from infinite (flat prior).

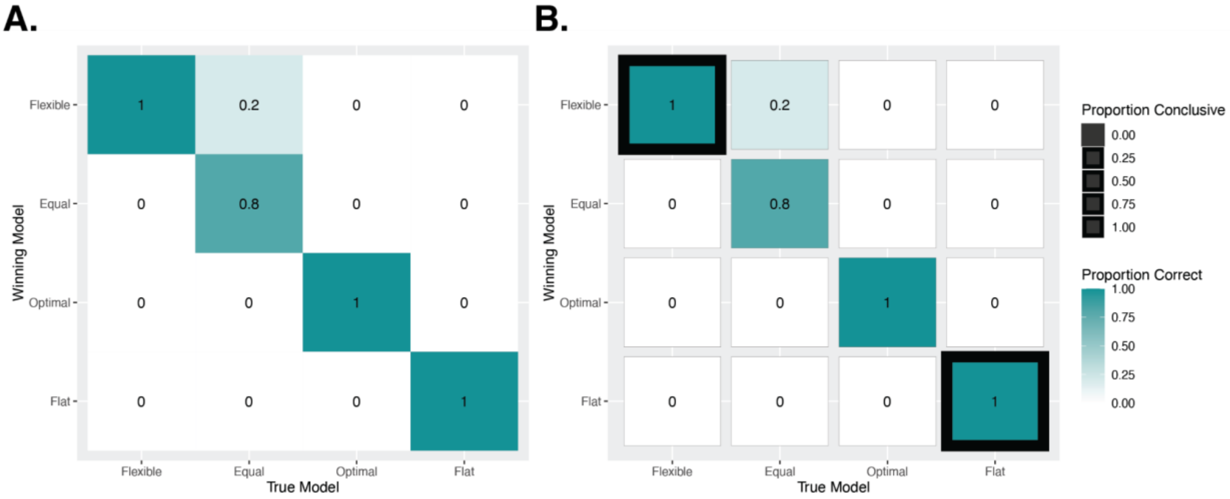

Figure S6. Model Recovery Analysis. A. Confusion Matrix. To test model recovery, we simulated data from each model and then compared the predictive performance of all models on that data. We consider the winning model to be that with the highest ELPD, and we consider this to be conclusive when the ELPD is higher by at least 4, and when the magnitude of the difference is at least 2 times the standard error of the difference. Columns correspond to the true generative model and rows correspond to the recovered model, with the cell colour indicating the proportion of repetitions in which that model (row) was the best fitting model, given the true model (column). Hence, cells that diverge from the diagonal indicate cases in which the model recovery got ‘confused’, or in other words, the best fitting model was not the true generative model. This analysis reveals good model recovery, with few cases of

model confusion, and a recovery accuracy of 95%. In the results shown here, data simulated from the Flexible Model were simulated using a parameter combination in which w_{choice} was larger than w_{conf} (w_{choice} = 2, w_{conf} = 1). However, we also performed a model recovery analysis using an instance of the Flexible Model in which this was reversed (w_{choice} = 1, w_{conf} = 2). This revealed no substantial differences - there was just one fewer 'conclusive' result (see B). For the Equal Model, we simulated data with the weighting parameter *w* set to 2 (for both decisions and confidence). For the Optimal Model, the weighting of prior information was optimal (1 in both decisions and confidence). For the Flat Prior Model, decisions and confidence were simulated without any use of prior information (equivalent to w_{choice} and w_{conf} being infinitely large). **B. Conclusive Results.** The model recovery performance shown in the confusion matrix in (A) is based on the winning criterion of having the highest ELPD. However, not all of these instances meet our more stringent criterion for a *conclusive* result: ELPD is higher by at least 4, and when the magnitude of the difference is at least 2 times the standard error of the difference. To show these cases, here we depict the proportion of model recovery repetitions in which the winning result was conclusive, by changing the border thickness. This reveals that the Flexible Model and the Flat Prior models are always the conclusive winners at recovering data generated from themselves, but the Equal and Optimal models are never conclusive in their superior recovery. However, importantly, the only time in which the Flexible Model was conclusively superior in terms of predictive performance was when data were simulated from that model, and hence when w_{conf} and w_{choice} were really different than optimal, different than each other, and not infinitely large, suggesting the models to be distinguishable in the required way. Source data are provided as a Source Data file.

Flexible Model - Experiment 3

Experiment 3 used an adapted version of the non-simplified model, in order to suit the instructed probabilistic priors and single decision task. The simplification was not needed, since there was only one decision and hence one internal signal on every trial, which significantly reduced model complexity and the number of free parameters.

Model Adaptations

The computation of the target decision variable and of explicit confidence followed the same formulae as the non-simplified model for Experiment 1 and 2. However, the computation of the prior did not come from an internal decision confidence but rather from the externally instructed prior probability. In order to implement the weighting parameters in the same way as the previous model, we converted the explicit prior probability to an equivalent internal signal using

$$r_{prior} = \phi^{-1}(P(R)_{explicit})$$

such that we could again use the following equations to compute the effect of the weighted prior in decisions and confidence:

$$\theta = -\frac{\sigma_{likelihood}}{w_{choice}\sigma_{prior}}|r_{prior}|$$

$$p(R)_{conf} = \Phi(\frac{|r_{prior}|}{b\ w_{conf}\ \sigma_{prior}})$$

Additionally, in Exp. 1 and 2, the matched conditions allowed us to fit the weighting parameters without interference from metacognitive noise. In the case of Exp. 3, without the matched conditions, we needed to account for metacognitive noise explicitly in the model fitting. To do so, we fit metacognitive noise based on a control condition without an informative prior (see below). This value was put into the full model fitting. We sampled the internal signal r_{target} for the confidence computation specifically, called r_{conf} below, from a distribution that took this total amount of noise at the metacognitive level into account:

$$r_{conf} \sim \mathcal{N}(s, \boldsymbol{\sigma}_{\text{meta}})$$

where s represents the stimulus, and $\boldsymbol{\sigma}_{\text{meta}}$ was the total noise at the metacognitive level, which could either reflect added metacognitive noise or a reduction of noise (eg. reflecting the case of M-Ratios above 1) compared to the first order internal noise.

The model fitting followed the same procedure as in Exp. 1 and 2, except that we added more noise (σ=0.025) between the predicted confidence (model_conf) and the observed confidence ratings, which corresponds to small uncertainties when using the confidence scale[2]. This was necessary for model convergence, likely due to the non-simplified implementation. The model was nevertheless able to well account for confidence.

Fitting Metacognitive Noise

We fit metacognitive noise for each participant using trials from the 50% (non-informative) prior condition of the main experimental task as well as the 180 trials of the control task (which included confidence in Exp. 3), giving a total of 280 trials. The equations for the modelled decisions and confidence on these control trials matched those described above in the full model, but with a prior of 0.5. We sampled the internal signal for the confidence computation, r_{conf}, from a distribution centred around the external stimulus s_{target}, with noise equal to $\boldsymbol{\sigma}_{\text{meta}}$ instead of the previously fit internal noise value, accounting for the potentially different noise at the metacognitive level. There were two free parameters of interest in this model: $\boldsymbol{\sigma}_{\text{meta,}}$ and b, which was still needed in order to capture confidence bias, as this could otherwise interfere with the fitting of $\boldsymbol{\sigma}_{\text{meta}}$. Lognormal priors were set on $\boldsymbol{\sigma}_{\text{meta,}}$ and b. Once fit, $\boldsymbol{\sigma}_{\text{meta}}$ was input to the full model in order to account for metacognitive noise, such that it could not interfere with the fitting of the other parameters. The confidence bias b fit in this stage was not used as the final interpreted b, which we fit again in the full model. This is because we assumed that it may differ slightly during the full task, and when including more trials across a longer experimental session.

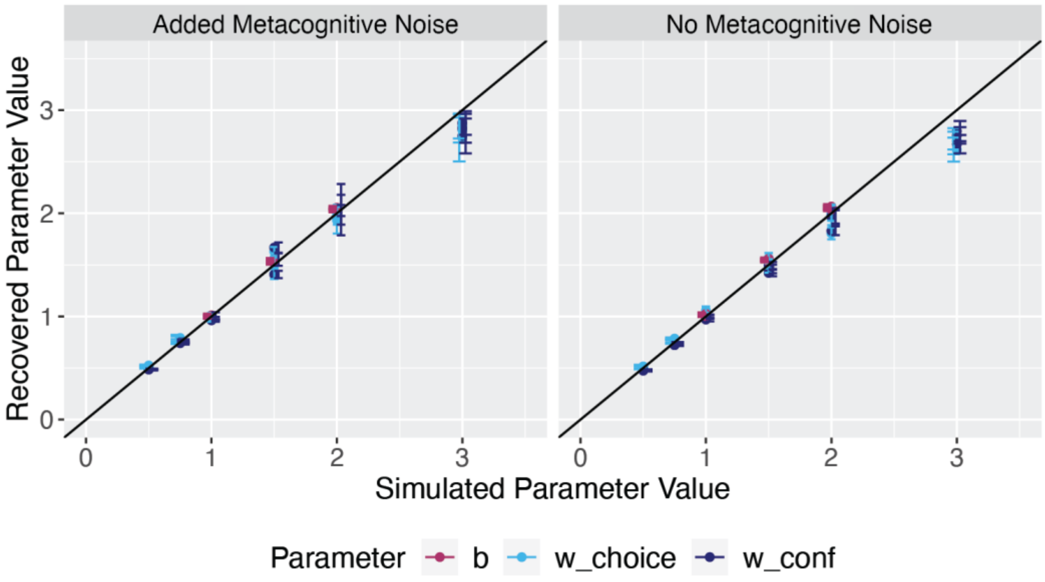

Figure S7. Experiment 3 Parameter Recovery Analysis. Parameter recovery after simulating from the model with rightward priors of 0.1, 0.2, 0.3, 0.4, 0.5, 0.6, 0.7, 0.8, and 0.9. The black line indicates perfect parameter recovery. This reveals good parameter recovery of the model. Points reflect the mean recovered parameter values across 10 repetitions of simulating data and fitting the model, and error bars reflect SEM across these repetitions. The left panel shows parameter recovery after simulating data with additional metacognitive noise, and then inputting this metacognitive noise to the model. Good parameter recovery indicates that the model can account for metacognitive noise without it interfering with the fitting of the other parameters. Source data are provided as a Source Data file.

Supplementary Discussion

Alternative Explanations

We now consider our findings (in particular from Exp. 1 and 2) in light of several other models or proposed features of decision and confidence behaviour that are central in the literature.

Metacognitive Noise

Several studies have shown that metacognitive noise is necessary to account for young healthy participants' confidence ratings[4,5]. We evaluated whether metacognitive noise could account for any of our findings. Here, we model metacognitive noise by adding noise to the internal signal just before computing confidence, in turn making confidence judgments noisier. As a result, confidence ratings track accuracy less sensitively. In the dual-decision paradigm, the rightward prior is susceptible to metacognitive noise, because it is itself based on an internal confidence computation. The strength of the rightward prior is equal to the point estimate of confidence on each trial. So, just as metacognitive noise makes confidence judgments noisier across trials, it will lead to more variable estimated prior strengths across

trials. In other words, the priors will sometimes be estimated as stronger or weaker than appropriate, making them less reflective of the correct priors. Note that in this task, the effect that metacognitive noise has on priors is different from the intuition, stemming from Bayesian integration, that noisier signal distributions at the metacognitive level will make the prior weaker overall. This is because the strength of the prior is based on the point estimate of confidence on a given trial, rather than the width of the Type 2 distributions. So, this poorly estimated prior will make target decision accuracy worse, but will not lead to consistently over- or underestimated priors, or to differences between conditions.

In order to further explore the effects of metacognitive noise, and to ensure that it cannot provide an alternative explanation for any of our findings, we consider three different possible implementations of metacognitive noise in our model:

(1) One possibility is that, in order to combine the prior and likelihood in a precision-weighted way, participants form an internal confidence/precision estimate about both the lead and target stimulus. In this case, both precision-estimates would get corrupted by the same metacognitive noise, hence this is referred to as 'Balanced Metacognitive Noise' below. This decreases target decision accuracy but does not cause a difference between conditions (Figure S8C).
(2) Another possibility is that the prior causes a shift in the decision criterion, and the target decision is then made by comparing the internal signal from the target stimulus to that criterion, without a precision-estimate of the target stimulus. Then, only the prior is corrupted by metacognitive noise, leading to noisier prior values across trials, while the likelihood stays less varied. This decreases target decision accuracy, since the estimated prior deviates somewhat from the true prior, but it does not cause a consistent over- or underestimation of the prior and hence does not interact with the conditions (Figure S8D).
(3) It is possible that, no matter how these computations occur, metacognitive noise only occurs in forming an explicit confidence rating. Then, metacognitive noise would only impact the target confidence and target decisions would not be impacted at all (Figure S8E).

None of these possible implementations can explain our decision level results, namely a difference in target decision accuracy between conditions (Figure S8A), which can instead be captured by an underweighting of the prior in decisions.

To further ensure that metacognitive noise does not interfere with our conclusions, we next showed that it cannot provide an alternative explanation for the asymmetry between the decision and confidence levels. To show this, we checked that, if the weighting were the same

in confidence as in the target decisions, adding metacognitive noise cannot produce the patterns found. So, we simulated w_{conf} equal to w_{choice} (2.17) with each of the different metacognitive noise implementations above (Figure S8C-E). None of these implementations can produce the confidence patterns that we see (Figure S8A), which can instead be captured by the asymmetrical weighting.

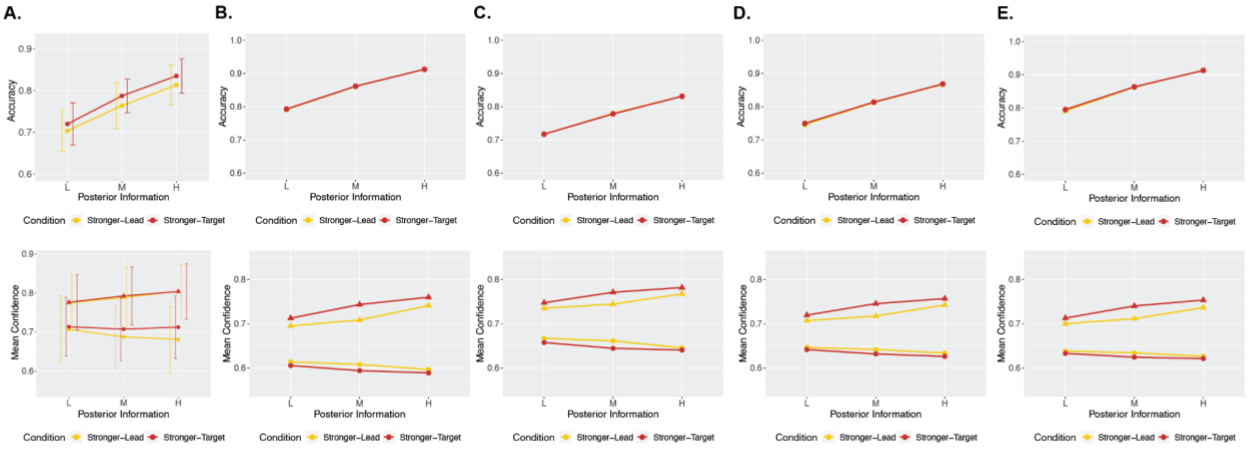

Figure S8. Metacognitive Noise Simulations. A. Observed Target Decision and Confidence Results. (Top) Decision level results showing target accuracy per condition and posterior information level. (Bottom) Confidence level results showing mean confidence following correct and incorrect trials per condition and posterior information level. Error bars capture standard deviation (SD) across participants (N=20). **B. Simulations with No Metacognitive Noise.** (Top) Decision level results given optimal weighting of the prior (w_{choice} = 1), with no metacognitive noise. This is used as a comparison for C-E, to show the effect of adding metacognitive noise. (Bottom) Confidence level results given the decision level result found (w_{choice} = 2.17) but equal weighting of the prior in confidence (w_{conf} = 2.17), and no metacognitive noise. The fit confidence bias (b = 2.18) was also used. This is used as a comparison for C-E to show the effect of adding metacognitive noise. **C. Simulations with Balanced Metacognitive Noise.** (Top) Decision level results given optimal prior weighting but with metacognitive noise in estimating the precision of both the prior and likelihood. (Bottom) Confidence level results given equal underweighting of the prior between decisions and confidence, and added metacognitive noise in estimating the precision of both the prior and likelihood. **D. Simulations with Metacognitive Noise Corrupting Prior Only.** (Top) Decision level results given optimal prior weighting but with metacognitive noise in estimating just the prior. (Bottom) Confidence level results given equal underweighting of the prior between decisions and confidence, and added metacognitive noise in estimating just the prior. **E. Simulations with Metacognitive Noise for Explicit Confidence Only.** (Top) Decision level results given optimal prior weighting but with metacognitive noise added only for explicit confidence, which does not impact the decision level. (Bottom) Confidence level results given equal underweighting of the prior between decisions and confidence, and added metacognitive noise just in explicit confidence. For all simulations shown here, substantial metacognitive noise was added, approximately equivalent to an MRatio of 0.5. Source data are provided as a Source Data file.

Lognormal Metacognitive Noise

Another open question in the literature is what form such metacognitive noise takes. Though most work has assumed Gaussian metacognitive noise, it has also recently been proposed that lognormally distributed noise better accounts for metacognitive ratings[6].

Lognormal metacognitive noise leads to more impact on extreme signals, or on higher confidence criteria. However, when investigating this metacognitive noise model, we similarly find it to lead to noisier ratings overall, but not consistent differences between our conditions in any of the above implementations, so it cannot account for our pattern of results.

Translation Noise

Because of the nature of the prior as an internal confidence value in the dual-decision task, it is possible that its computation exists in a Type 2, metacognitive processing space. If this is the case, it may also be that in order to make use of that prior in the target decision, it must be translated back to the Type 1 decision space. This translation could itself also be corrupted by a "translation" noise, similar to the metacognitive noise that occurs when moving from the Type 1 to the Type 2 space. Translation noise will have the same impact as metacognitive noise, making the prior estimation noisier and less reflective of the true prior, but not consistently too weak or too strong. Simulations (not included here) have revealed that, exactly as with adding metacognitive noise, (a) target decision accuracy decreases and (b) confidence patterns get less informative following correct vs incorrect target decisions, but critically it does not interact with our conditions. We conclude that it cannot provide an alternative explanation for our results.

Confirmatory Evidence Accumulation

We find that participants do not combine both sources of evidence (prior and likelihood) optimally when forming their target decision. This is in line with previous work that has found non-additive or biased combinations of evidence. For example, several studies have found a confirmation bias in the way that evidence accumulates over time, with later evidence being biased towards supporting the decision already made, even if that decision was internal[7–9]. If that occurs in our paradigm, we would expect the second decision to be biased towards confirming the first decision. Although this is not in line with the rule we gave participants, it is possible that they gathered evidence in this way. To assess this, we investigated whether participants performed better when the direction of the target stimulus matched the lead, which would be expected if they are biased to confirm their lead decisions. This revealed participants *not* to have such a confirmatory bias, and in fact to have a small bias in the opposite direction, or a slight repulsion effect, with participants being more likely to choose right on targets following a leftward lead decision. So, this confirmatory evidence accumulation does not explain our pattern of results. Additionally, at the confidence level this confirmatory behaviour would lead to higher confidence following confirmatory decisions, compared to opposing decisions (Figure S9B), which we do not find in the data (Figure S9A). Importantly, though we

find a repulsion effect at the decision level, the confidence patterns also do not fit what would be expected of an opposing choice bias, which produces lower confidence following confirmatory decisions, compared to opposing decisions (Figure S9C). Hence, this opposing choice effect also cannot capture our pattern of results. However, we consider the repulsion effect further in the section below, as it may stem from motion aftereffects, which are possible from our stimuli.

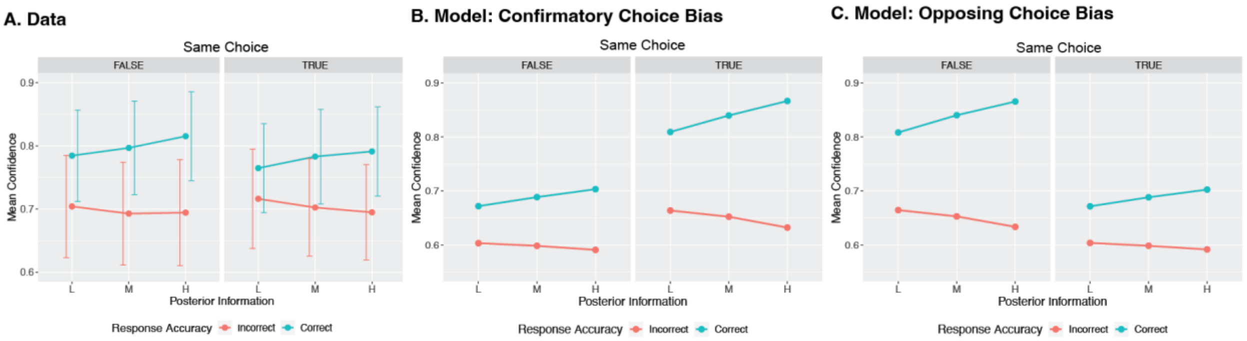

Figure S9. Predicted Confidence with Confirmatory and Opposing Choice Biases. A. Data. The mean observed confidence following correct and incorrect trials per posterior information level, split by whether the target decision was the same as or was different from the lead decision. Error bars indicate SD across all participants (N=21). **B. Simulated Confidence with Confirmatory Choice Bias.** Simulated confidence with a confirmatory bias such that target evidence is biased to be more strongly in line with the lead decision. This leads to higher confidence (following both correct and incorrect trials) when the target decision is the same as (confirms) the lead decision. **C. Simulated Confidence with Opposing Choice Bias.** Simulated confidence with an opposing choice bias such that target evidence is biased away from the direction of the lead decision. This leads to higher confidence (following both correct and incorrect trials) when the target decision goes against (opposes) the lead decision. Source data are provided as a Source Data file.

We suggest that the difference in results here compared to the previous studies that found such a confirmation bias is due to the structure of the dual-decision task used, in which participants were explicitly informed of the rule that the direction of the target stimulus depended on their lead decision response accuracy. If the task had not given explicit instructions regarding how to combine the evidence from the two stimuli, they may have acted in a more confirmatory way.

Motion Aftereffects

Given the dot motion stimuli that we used, and the finding of a repulsion effect in decisions, we consider the possibility that there are motion aftereffects that can contribute to this repulsion bias. To assess this, we needed to disentangle this effect from the effects of how participants used the informative priors. So, we used our control task (180 trials per participant) in which the stimuli were the same as in the main task except their directions were each independent and 50/50 left versus right. This indeed revealed a weak repulsion bias. To

quantify it, we modelled and fit the strength of motion aftereffects in the control task. Then, in order to further investigate how they may interact with our findings, we simulated responses to the main task with motion aftereffects of the fit strength (also incorporating the fit decision bias and internal noise for each participant), and with optimal weighting of the prior in decisions and confidence. This way, we tested whether aftereffects alone could account for our pattern of results. We found that motion aftereffects do not produce differences between the conditions, and hence cannot alone account for the findings (Figure S10). We suggest that the aftereffects likely remained very weak due to the long interstimulus interval, which had a median over 2000 ms in both the control and main task, at which point motion aftereffects have been shown to decrease dramatically[10]. We note that the above analyses were based on Exp. 1, but the fit strength of the aftereffects were even weaker in Exp. 2.

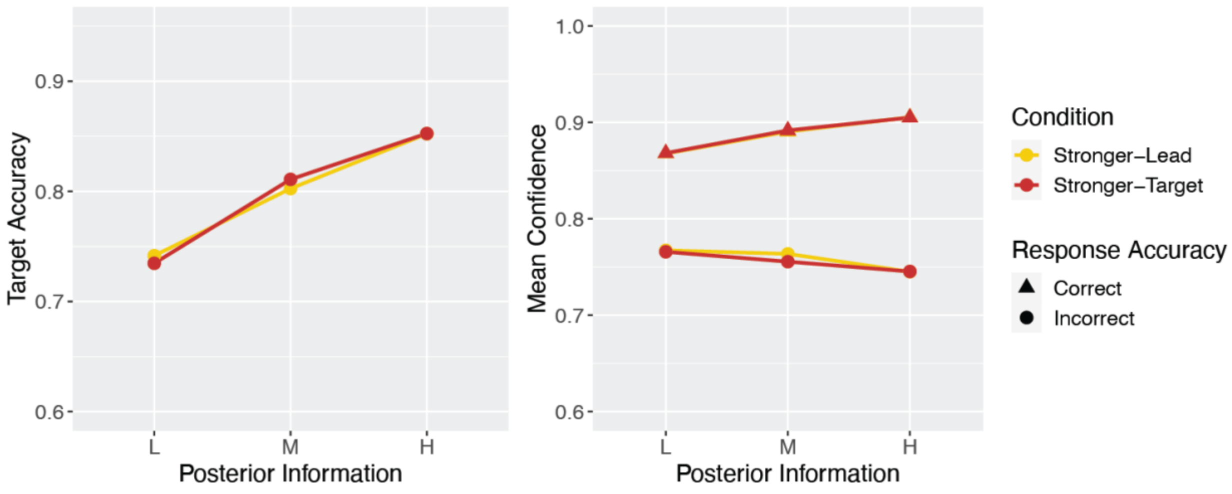

Figure S10. Motion Aftereffect Simulations. Here we show the simulated target accuracy and mean confidence per condition and posterior information level, including simulated motion aftereffects and optimal prior weighting. We simulated using the true stimulus strengths of the main task, fit decision bias and internal noise per participant, as well as the overall fit motion aftereffect strength. The motion aftereffect strength was fit to the control task trials. This reveals that the motion aftereffects found are very weak and cannot explain the differences we see between conditions, hence they cannot explain our pattern of results. Source data are provided as a Source Data file.

Confidence Leak

The use of a prior that is itself an internal confidence value leads to the possibility that there is an impact of the internal confidence on the explicit confidence, beyond the prior strength. This would be in line with the finding that confidence can "leak" from one trial to the next[11]. Here, it is possible that internal confidence from the lead decision likewise biases the explicit confidence. This would not, however, impact the weighting of the prior in the target decision, so it could not explain the underweighted prior observed at the decision level. In confidence, this confidence leak model predicts that the higher internal lead decision confidence in the Stronger-Prior condition would leak to produce more extreme target decision

confidence reports. However, given the underweighted prior in decisions, simulations from a confidence leak model showed that the effects on confidence cannot explain the asymmetry between decisions and confidence that we find, either at moderate values based on previous findings[11] (Figure S11B-C), nor at extreme values (Figure S11D). This also cannot capture the pattern of results when it interacts with metacognitive noise (Figure S11C-D). Confidence leak was simulated by setting the final explicit confidence rating to be a weighted average between the target confidence (before any leak) and the internal lead confidence, and strength of the leak was manipulated by adjusting the relative weight of lead confidence in this average.

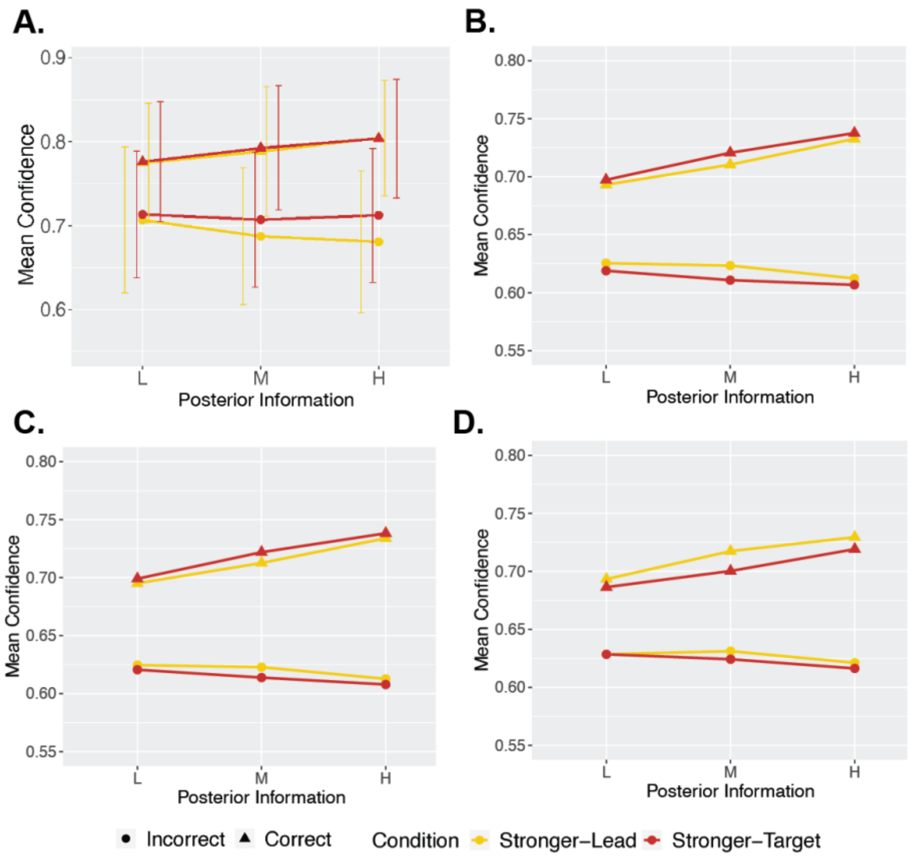

Figure S11. Confidence Leak Simulations. Simulations of confidence patterns given equal underweighting of priors in decisions and confidence (w_{choice} and w_{conf} = 2.17), with an additional confidence leak effect. **A. Data.** The true confidence patterns for correct versus incorrect trials per condition and posterior information level. Error bars indicate SD across all participants (N=20). **B. Moderate Confidence Leak.** Simulations of confidence without metacognitive noise, and with a moderate confidence leak strength such that one unit of confidence increase on the lead decision predicts an increase of target confidence of approximately 0.25. **C. Moderate Confidence Leak with Metacognitive Noise.** Simulations of confidence with the same amount of confidence leak as in (B), but with added metacognitive noise. **D. Extreme Confidence Leak.** Simulations demonstrating an extreme confidence leak, to show the direction in which this shifts results. This also includes the same metacognitive noise as in (C). Together, this reveals that confidence leak cannot account for the pattern of results – even in interaction with metacognitive noise or at extreme values – which can instead be captured by asymmetrical weighting of prior information. Source data are provided as a Source Data file.

Prior Incongruence Analysis

Here, we consider the results split by trials in which the likelihood was congruent versus incongruent with the prior. We assume that confidence ranged from 50-100%, and hence the rightward prior also ranged from 50-100%. This means that leftward target stimuli would typically be incongruent with the prior. We can then examine this subset of prior-incongruent trials, which were trials with leftward target stimuli, or in other words, with incorrect lead decisions. Out of these prior-incongruent trials, the strongest incongruence would occur on trials with the strongest rightward prior, which occur with high lead decision confidence, despite the incorrect lead decision. Though it is difficult to identify incorrect lead decisions that have high confidence, since we do not have explicit lead confidence ratings, the folded-X pattern of the Bayesian confidence model predicts these to occur at the lowest stimulus intensity. Together, we assume the prior-incongruent trials to be ones with incorrect lead decisions, and we expect stronger incongruency (and therefore lower accuracy) with lower stimulus intensities. This is also shown in model predictions in Figure S12B, with lower target decision accuracy for lower stimulus intensities for prior-incongruent trials. We find the data to go in line with these predictions (Figure S12A).

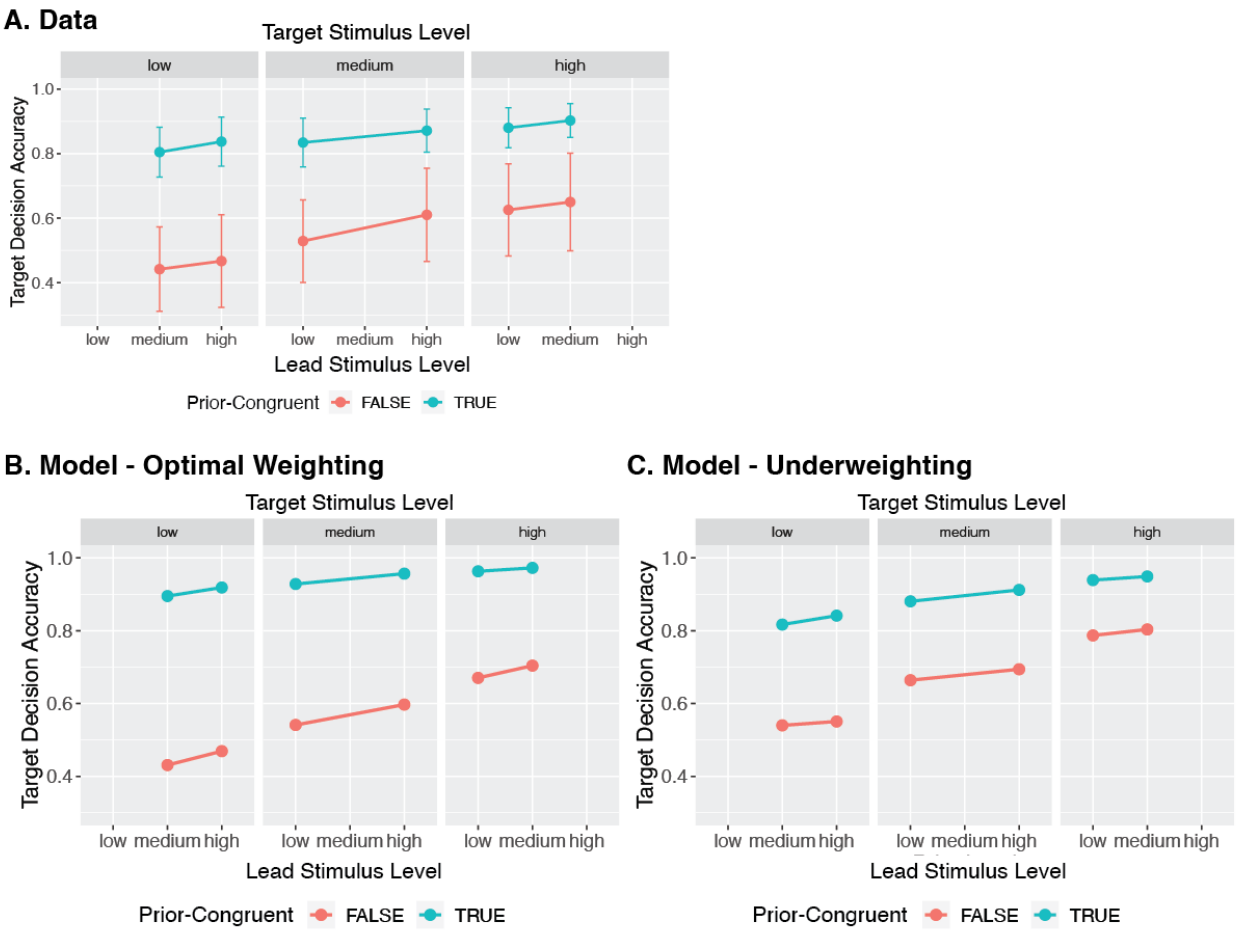

Figure S12. Target Accuracy on Prior-Incongruent vs Prior-Congruent Trials. A. Observed Target Accuracy. Mean observed target decision accuracies per lead stimulus level at each target stimulus

level, which are split into panels. Accuracies are shown based on prior-congruent (blue) versus prior-incongruent trials (red), with prior-incongruent trials referring to leftward target stimuli, or incorrect lead decision trials. Error bars capture standard deviation of accuracies across participants (N=21). **B. Simulated Target Accuracy with Optimal Prior Weighting.** Simulated target decision accuracies given optimal weighting of the prior (w_{choice} = 1), shown based on prior-congruent (blue) versus prior-incongruent trials (red). **C. Simulated Target Accuracy with Underweighting of Priors.** Simulated target decision accuracies given optimal weighting of the prior (w_{choice} = 2), shown based on prior-congruent (blue) versus prior-incongruent trials (red). Source data are provided as a Source Data file.

Further, we qualitatively compared the data to model predictions on prior-congruent versus prior-incongruent trials, given optimal weighting and underweighting of the prior. On prior-congruent trials, participants did not perform as well as would be predicted of an optimal observer, suggesting that they are underusing the informative prior on those trials, in line with what is shown in the “Underweighting” model simulations (Figure S12C). However, on prior-incongruent trials, participants seem to ‘stick to’ the rightward prior that they form, although this then goes against the likelihood, which actually keeps their accuracy low, close to that of the “Optimal” model for incongruent trials. Interestingly, this might suggest that the underweighting of the prior in target decisions that we find is driven more by prior-congruent trials underusing the prior, rather than by prior-incongruent trials.

We then consider confidence predictions following prior-congruent versus prior-incongruent target stimuli. With prior-incongruent target stimuli (aka leftward target stimuli), correct target decisions were prior-incongruent target decisions (aka they chose “left”). Incorrect target decisions were prior-congruent target decisions (aka they chose “right” despite the leftward stimulus). This means that when the target stimulus is very weak and participants are presumably unsure about the target stimulus itself, confidence is actually expected to be higher following incorrect decisions, since those go in line with the prior (Figure S13B, left panel). We found target confidence to follow this pattern for incongruent trials (Figure S13A in red). However, we found that pattern for all levels of the target stimulus, not just the low precision level. To investigate what this suggests about the prior weighting, we simulated data with both optimal prior weighting and with prior weighting that is stronger in confidence than in decisions, in line with our other results, and explored confidence patterns for prior-incongruent versus prior-congruent trials. This reveals that stronger weighting of the prior in confidence compared to decisions can cause what we see in the data for prior-incongruent trials: higher confidence following incorrect decisions on prior-incongruent trials, at all levels of target stimuli used (Figure S13C). This adds additional evidence to support our conclusion of stronger use of priors in confidence compared to decisions.

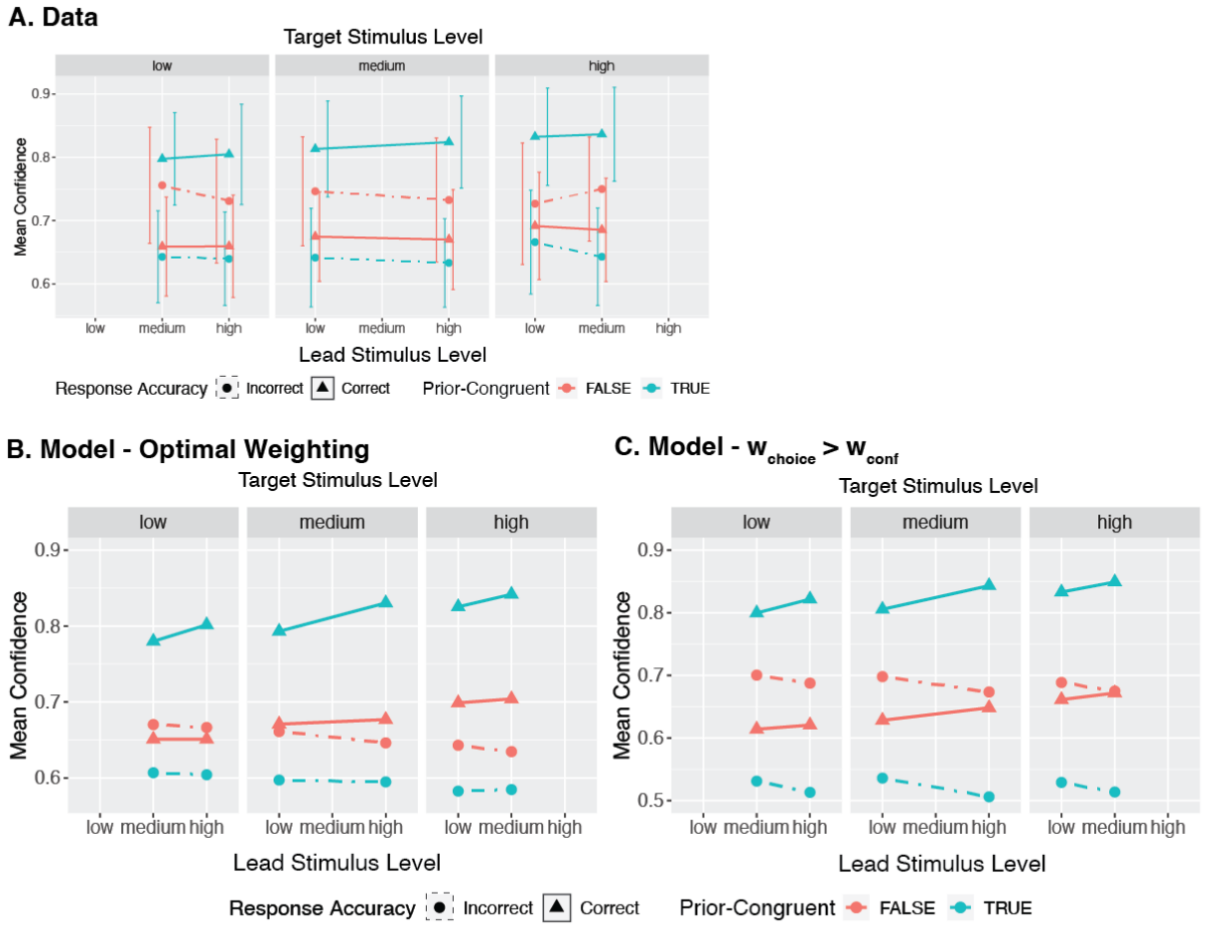

Figure S13. Confidence on Prior-Incongruent vs Prior-Congruent Trials. A. Observed Confidence. Observed mean confidence following correct and incorrect trials, shown per lead stimulus level at each target stimulus level, which are split into panels. Confidence is also divided by prior-congruent trials (blue) and prior-incongruent trials (red). For prior-incongruent trials, note that correct decisions involve decisions that are in line with the likelihood but not the prior, and incorrect decisions involve decisions that are in line with the prior but not likelihood. Error bars capture standard deviation of mean confidence across participants (N=21). **B. Simulated Confidence with Optimal Prior Weighting.** Confidence patterns for prior-congruent versus prior-incongruent trials, from simulated data with optimal prior weighting (w_{choice} and w_{conf} = 1). **C. Simulated Confidence with Stronger Prior Weighting in Confidence than in Decisions.** Confidence patterns for prior-congruent versus prior-incongruent trials, from simulated data with stronger prior weighting in confidence compared to decisions (w_{conf} = 1, w_{choice} = 2). Source data are provided as a Source Data file.

Supplementary References

1. Lisi, M., Mongillo, G., Milne, G., Dekker, T. & Gorea, A. Discrete confidence levels revealed by sequential decisions. *Nat. Hum. Behav.* **5**, 273–280 (2021).
2. Fleming, S. M., van der Putten, E. J. & Daw, N. D. Neural mediators of changes of mind about perceptual decisions. *Nat. Neurosci.* **21**, 617–624 (2018).
3. Vehtari, A. *et al.* loo: Efficient leave-one-out cross validation and WAIC for Bayesian models. (2022).
4. Shekhar, M. & Rahnev, D. The sources of metacognitive inefficiency. *Trends Cogn. Sci.* **25**, 12–23 (2021).
5. Bang, J. W., Shekhar, M. & Rahnev, D. Sensory Noise Increases Metacognitive Efficiency. *J. Exp. Psychol. Gen.* **148**, (2018).
6. Shekhar, M. & Rahnev, D. The nature of metacognitive inefficiency in perceptual decision making. *Psychol. Rev.* **128**, 45–70 (2021).
7. Rollwage, M. *et al.* Confidence drives a neural confirmation bias. *Nat. Commun.* **11**, 2634 (2020).
8. Talluri, B. C. *et al.* Choices change the temporal weighting of decision evidence. *J. Neurophysiol.* **125**, 1468–1481 (2021).
9. Talluri, B. C., Urai, A. E., Tsetsos, K., Usher, M. & Donner, T. H. Confirmation Bias through Selective Overweighting of Choice-Consistent Evidence. *Curr. Biol.* **28**, 3128-3135.e8 (2018).
10. Hershenson, M. Duration, time constant, and decay of the linear motion aftereffect as a function of inspection duration. *Percept. Psychophys.* **45**, 251–257 (1989).
11. Rahnev, D., Koizumi, A., McCurdy, L. Y., D'Esposito, M. & Lau, H. Confidence Leak in Perceptual Decision Making. *Psychol. Sci.* **26**, 1664–1680 (2015).
