# Supplementary material for: Prior information differentially affects discrimination decisions and subjective confidence reports
Source: Nat Commun. 2023 Sep 6;14:5473. doi: 10.1038/s41467-023-41112-0 (PMC10482953; doi:10.1038/s41467-023-41112-0)
Supplement: Supplementary file 3 — Reporting Summary [file 41467_2023_41112_MOESM3_ESM.pdf]

nature portfolio

Corresponding author(s): Marika Constant
Last updated by author(s): July 10, 2023

Reporting Summary

Nature Portfolio wishes to improve the reproducibility of the work that we publish. This form provides structure for consistency and transparency in reporting. For further information on Nature Portfolio policies, see our Editorial Policies and the Editorial Policy Checklist.

Statistics

For all statistical analyses, confirm that the following items are present in the figure legend, table legend, main text, or Methods section.

n/a	Confirmed	
☐	☒	The exact sample size (*n*) for each experimental group/condition, given as a discrete number and unit of measurement
☐	☒	A statement on whether measurements were taken from distinct samples or whether the same sample was measured repeatedly
☐	☒	The statistical test(s) used AND whether they are one- or two-sided<br>*Only common tests should be described solely by name; describe more complex techniques in the Methods section.*
☒	☐	A description of all covariates tested
☐	☒	A description of any assumptions or corrections, such as tests of normality and adjustment for multiple comparisons
☐	☒	A full description of the statistical parameters including central tendency (e.g. means) or other basic estimates (e.g. regression coefficient) AND variation (e.g. standard deviation) or associated estimates of uncertainty (e.g. confidence intervals)
☐	☒	For null hypothesis testing, the test statistic (e.g. F, t, r) with confidence intervals, effect sizes, degrees of freedom and P value noted<br>*Give P values as exact values whenever suitable.*
☐	☒	For Bayesian analysis, information on the choice of priors and Markov chain Monte Carlo settings
☒	☐	For hierarchical and complex designs, identification of the appropriate level for tests and full reporting of outcomes
☐	☒	Estimates of effect sizes (e.g. Cohen's *d*, Pearson's *r*), indicating how they were calculated

Our web collection on statistics for biologists contains articles on many of the points above.

Software and code

Policy information about availability of computer code

Data collection	Data was collected using computer-based experiment developed in HTML/Javascript/CSS to run in the browser using Google Chrome (version 94.0.4606.71). JATOS (version 3.7.4) was used to store the result data.
Data analysis	Data was analyzed using code written in R 4.1.2 and using STAN (version 2.21.0). The packages 'lme4' (version 1.1.27.1), 'bayestestR' (version 0.13.0), 'cmdstanr' (version 0.5.1), 'loo' (version 2.4.1) and 'brms' (version 2.16.3) were used in R for data analysis. Custom code for data analysis is publicly available at: https://gitlab.com/MarikaConstant/priors-in-confidence.

For manuscripts utilizing custom algorithms or software that are central to the research but not yet described in published literature, software must be made available to editors and reviewers. We strongly encourage code deposition in a community repository (e.g. GitHub). See the Nature Portfolio guidelines for submitting code & software for further information.

Data

Policy information about availability of data

All manuscripts must include a data availability statement. This statement should provide the following information, where applicable:

- Accession codes, unique identifiers, or web links for publicly available datasets
- A description of any restrictions on data availability
- For clinical datasets or third party data, please ensure that the statement adheres to our policy

The raw experimental data generated in these experiments have been deposited in a public repository on Zenodo under https://doi.org/10.5281/zenodo.8131976.

Research involving human participants, their data, or biological material

Policy information about studies with human participants or human data. See also policy information about sex, gender (identity/presentation), and sexual orientation and race, ethnicity and racism.

Reporting on sex and gender	Neither sex nor gender played a role in our research questions and we did not consider sex or gender in the study design. Gender was determined based on free-form, optional self-reporting. In Experiment 1, out of our 21 included participants, 10 self-reported as male and 11 self-reported as female. In Experiment 2, out of our 25 included participants, 9 self-reported as male, 15 self-reported as female, and 1 chose not to specify. In Experiment 3, out of our 23 tested participants, 4 self-reported as male and 19 self-reported as female. No gender-based analysis was performed as this did not factor in to our research questions.
Reporting on race, ethnicity, or other socially relevant groupings	No socially constructed or socially relevant categorization variables played a role in our research questions or study design, or were used in our manuscript.
Population characteristics	See below.
Recruitment	Participants were recruited through the participant pools at the Humboldt-Universität zu Berlin and Berlin School of Mind and Brain, and through word-of-mouth. It is possible that there are selection biases such as a bias towards students, or participants that choose to attend this type of experiment for other reasons, but we do not suspect any impact of such biases on our results.
Ethics oversight	The ethics committee of the Institute of Psychology at the Humboldt-Universität zu Berlin approved the study (Nr. 2021-47), which conformed to the Declaration of Helsinki.

Note that full information on the approval of the study protocol must also be provided in the manuscript.

Field-specific reporting

Please select the one below that is the best fit for your research. If you are not sure, read the appropriate sections before making your selection.

☐ Life sciences ☒ Behavioural & social sciences ☐ Ecological, evolutionary & environmental sciences

For a reference copy of the document with all sections, see nature.com/documents/nr-reporting-summary-flat.pdf

Behavioural & social sciences study design

All studies must disclose on these points even when the disclosure is negative.

Study description	The study consisted of three quantitative experiments. All three experiments used a within-subjects design.
Research sample	21 participants were included in Experiment 1 (mean age = 26, for gender see above), 25 participants were included in Experiment 2 (mean age = 25, for gender see above), and 20 participants were included in Experiment 3 (mean age = 24, for gender see above). Participants were tested in Berlin. We did not take specific measures to ensure a representative sample. We chose to use a convenience sample of healthy, young participants because our research question did not call for a specific demographic or population.
Sampling strategy	We used a convenience sample and the sample sizes for the first two experiments were pre-registered. The sample size for the first experiment was based off of previous work that used similar methods. The main analysis and task design were similar to that of Lisi et al. (2020), with n=15 in a first experiment and n=16 in a replication. The hypothesis and modeling approach was also conceptually similar to that of Aitchison et al. (2015), with n=26. The sample size for the second experiment was based off of a power analysis from Experiment 1, with which we set a stopping rule. The sample size for the third experiment was not pre-registered, but was based directly off of the first two experiments. Bayesian statistics also indicated that the sample sizes were sufficient to provide substantial evidence either for the null or alternative hypotheses of interest.<br><br>Lisi, M., Mongillo, G., Milne, G., Dekker, T. & Gorea, A. Discrete confidence levels revealed by sequential decisions. Nat. Hum. Behav. 5, 273–280 (2021).<br>Aitchison, L., Bang, D., Bahrami, B. & Latham, P. E. Doubly Bayesian Analysis of Confidence in Perceptual Decision-Making. PLOS Comput. Biol. 11, e1004519 (2015).
Data collection	The data was collected on a laptop computer, using keyboard and mouse responses. Only the experimenter and participant were present in the room during the experiment. The experimenter was not blind to the conditions or study hypotheses during data collection.
Timing	Data collection for Experiment 1 took place between October and December 2021. Data collection for Experiment 2 took place between August and September 2022. Data collection for Experiment 3 took place between February and March 2023.
Data exclusions	For Experiment 1 and 2, participants were excluded from analysis if they did not meet our pre-registered inclusion criteria (based on the basic manipulations working as intended, and basic understanding of the task), either after the first session, or after the two

combined sessions. In Experiment 1, 12 participants were excluded for these reasons after the first session, and a further 4 were excluded after the second session. In Experiment 2, 5 were excluded after the first session, and 1 further was excluded after the second session. Individual trials were also removed prior to analysis based on pre-registered exclusion criteria (extreme reaction times). In Experiment 3, we determined a priori that participants would be excluded if they failed to demonstrate a basic understanding of the task (which we defined to occur if they had lower performance in the condition with the most information than the condition with the least information). 3 participants were excluded for this reason.

Non-participation: One participant dropped out after the first session due to illness.

Randomization: Participants were not allocated into experimental groups as this was a within-subjects design.

Reporting for specific materials, systems and methods

We require information from authors about some types of materials, experimental systems and methods used in many studies. Here, indicate whether each material, system or method listed is relevant to your study. If you are not sure if a list item applies to your research, read the appropriate section before selecting a response.

Materials & experimental systems

n/a	Involved in the study
☒	☐ Antibodies
☒	☐ Eukaryotic cell lines
☒	☐ Palaeontology and archaeology
☒	☐ Animals and other organisms
☒	☐ Clinical data
☒	☐ Dual use research of concern
☒	☐ Plants

Methods

n/a	Involved in the study
☒	☐ ChIP-seq
☒	☐ Flow cytometry
☒	☐ MRI-based neuroimaging
